# Correlating the 3D Morphology of Polymer-Based Battery Electrodes with Effective Transport Properties

**DOI:** 10.1021/acsami.4c17522

**Published:** 2024-11-21

**Authors:** Benedikt Prifling, Lukas Fuchs, Aigerim Yessim, Markus Osenberg, Melanie Paulisch-Rinke, Philip Zimmer, Martin D. Hager, Ulrich S. Schubert, Ingo Manke, Thomas Carraro, Volker Schmidt

**Affiliations:** 1 Institute of Stochastics, Ulm University, 89069 Ulm, Germany; 2 Institute of Modelling and Computational Science, Applied Mathematics, Helmut-Schmidt-Universität/Universität der Bundeswehr Hamburg, 22043 Hamburg, Germany; 3 Institute of Applied Materials, Helmholtz-Zentrum Berlin für Materialien und Energie, 14109 Berlin, Germany; 4 Laboratory of Organic and Macromolecular Chemistry (IOMC), Friedrich Schiller University Jena, 07743 Jena, Germany; 5 Center for Energy and Environmental Chemistry (CEEC), Friedrich Schiller University Jena, 07743 Jena, Germany; 6 Helmholtz Institute for Polymers in Energy Applications Jena (HIPOLE Jena), 07743 Jena, Germany

**Keywords:** polymer-based battery, electrode, FIB-SEM tomography, nanostructure, statistical image analysis, effective charge transport, structure−property relationship

## Abstract

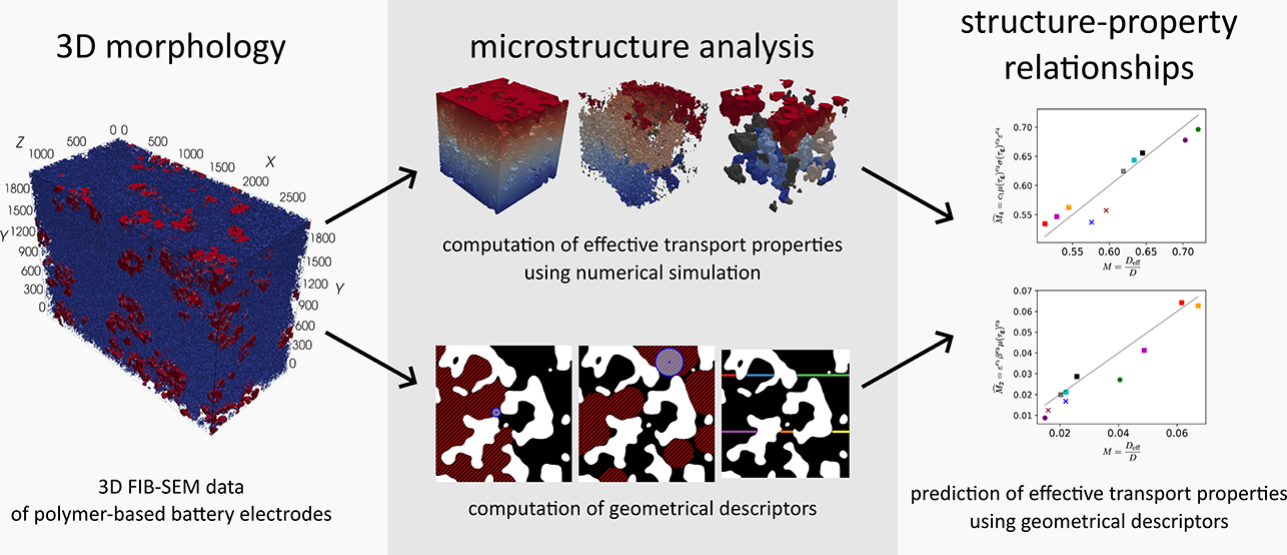

Polymer-based batteries represent a promising candidate for next-generation batteries due to their high power densities, decent cyclability, and environmentally friendly synthesis. However, their performance essentially depends on the complex multiscale morphology of their electrodes, which can significantly affect the transport of ions and electrons within the electrode structure. In this paper, we present a comprehensive investigation of the complex relationship between the three-dimensional (3D) morphology of polymer-based battery electrodes and their effective transport properties. In particular, focused ion beam scanning electron microscopy (FIB-SEM) is used to characterize the 3D morphology of three polymer-based electrodes which differ in material composition. The subsequent segmentation of FIB-SEM image data into active material, carbon-binder domain and pore space enables a comprehensive statistical analysis of the electrode structure and a quantitative morphological comparison of the electrode samples. Moreover, spatially resolved numerical simulations allow for computing effective properties of ionic and electronic transport. The obtained results are used for establishing analytical regression formulas which describe quantitative relationships between the 3D morphology of the electrodes and their effective transport properties. To the best of our knowledge, this is the first time that the 3D structure of polymer-based battery electrodes is quantitatively investigated at the nanometer scale.

## 1 Introduction

The recent technological progress regarding electric vehicles, portable devices and consumer electronics leads to increasingly demanding requirements for state-of-the-art batteries. Nowadays, the most commonly used type of batteries are lithium-ion batteries due to their low self-discharge rate, high power density and decent energy density.^1,2^ However, ecological and environmental aspects are of major importance with regard to next-generation battery technologies. In particular, organic active materials have the potential to overcome the disadvantages of classical lithium-ion batteries, namely a limited availability of the raw materials, a high toxicity and detrimental effects on the environment.^3−5^ Moreover, polymer-based batteries with organic active materials exhibit high-rate capabilities^6^ and can be realized in a flexible design,^7^ which enables the usage of polymer-based battery electrodes for small portable devices with a low energy consumption. In particular, a Ragone plot can be used to set the polymer-based thin film technology into context with other energy storage systems such as the dominating lithium ion technology and supercapacitors.^8,9^ Comparing polymer-based batteries, supercapacitors and the classical lithium-ion technology, the latter offers the highest energy density, whereas supercapacitors enable the highest power density. In view of the Ragone plot, polymer-based batteries are situated in between those two technologies, which makes them - among others - favorable for acting as dampening element in hybrid storage systems extenuating high charging rates with a reasonable energy density. In particular, poly(2,2,6,6-tetramethyl-4-piperinidyl-*N*-oxyl methacrylate) (PTMA) is one of the most investigated redox-active polymer since the early work at the beginning of the 2000s.^10,11^ This pioneering work set the basis for research into organic materials for energy storage solutions. The specific properties of PTMA, such as its high redox activity and stability, attracted the attention of researchers and industry to this technology and led to a growing interest in its commercial applications. In 2012 NEC tried to commercialize the first organic radical battery to power computers in case of power failure to prevent data loss.^12^ Recently, Evonik sold their material technology TAeTTOOz to InnovationLab.^13^ In general, the aim of these technologies is the development of a printable flexible polymer battery. For this purpose, quantifying relationships between the morphology of polymer-based battery electrodes and the resulting electrochemical performance is crucial. More precisely, a quantitative analysis of ionic and electronic transport processes in battery electrodes based on organic materials is critical since these are likely to be a limiting factor for the cell performance as in the case of classical lithium-ion batteries.^14−21^

The PTMA-based electrodes discussed here are composed of PTMA, a binder material, and SuperP, a conductive carbon black added to improve electronic conductivity (see Section 2.1 for more details on the material composition). A recent quantitative analysis of the 3D structure of this type of polymer-based electrode, conducted using synchrotron tomography, revealed a significant impact of the manufacturing processes on the resulting electrode microstructure.^22^ The present paper realizes a further step toward a systematic analysis of the 3D morphology of polymer-based electrodes, using focused ion beam scanning electron microscopy (FIB-SEM). To the best of our knowledge, this is the first time that the three-dimensional structure of this kind of battery electrodes is quantitatively investigated with such a high resolution. In particular, this goes far beyond the use of conventional 2D SEM as described in (23), since imaging via 2D SEM only allows a qualitative morphological analysis, whereas the 3D FIB-SEM tomography as applied in the present study enables a detailed analysis of the three-dimensional electrode microstructure.

Transport processes of charged particles (ions and electrons) in the porous electrodes play a central role in the overall electrochemical processes occurring in charging and discharging of the batteries. These processes take place at different spatial scales, each providing insights into the way ions and electrons move through the electrode. These processes can be described at three different scales: at the molecular level, where the interactions within the PTMA matrix are crucial; at the mesoscale, where the porous microstructure of the electrode plays a central role; and at the macroscale, where the effective transport properties determine the overall behavior of the battery.

At the molecular level, transport is determined by interactions between ions, electrons, and the polymer matrix. In PTMA, redox-active TEMPO units enable electron hopping in redox reactions, while ion transport is influenced by factors such as polymerization and cross-linking. This scale is critical to underst the fundamental interactions that determine the intrinsic electrochemical properties that define the performance limits of the material. Although understanding the molecular scale is essential for optimizing ion mobility and electron conductivity, empirical measurements can determine key parameters such as intrinsic ionic diffusivity, electronic conductivity, and reaction kinetics that can be used to model the electrode behavior at the macroscopic scale. Techniques such as electrochemical impedance spectroscopy (EIS) and galvanostatic cycling can be used to determine those parameters.

At the mesoscale, the electrode is treated as a porous medium with transport processes occurring within its microstructure. Charged particles move through it, with ions diffusing through the electrolyte and electrons conducting through the solid phase. The electronic conduction is dominated by the transport through the conductive additives since the PTMA has a lower intrinsic conductivity. The mesoscale provides an understanding of how factors such as pore size, tortuosity and phase connectivity influence transport efficiency. The concept of tortuosity generally refers to the complexity and length of the paths that ions and electrons must travel through the porous structure.^24^ These structural features determine how easily ions can diffuse through the electrolyte-filled pores and how well electrons can pass through the solid phase. This scale is critical to how effectively the electrode can support electrochemical reactions and how well it balances ion and electron transport, which ultimately affects the overall performance and current output of the battery. Mesoscale analysis plays a key role in optimizing microstructures to enhance battery efficiency and performance, often using physics-based models like the pseudo two-dimensional (P2D) electrochemical model, widely applied in conventional lithium-ion batteries following the foundational work of Doyle, Fuller, and Newman.^25−31^ More recently, a modified Doyle-Fuller-Newman model has been developed for PTMA-based battery electrodes to describe charge transport processes in dual-ion batteries.^32,33^

At the macroscale, the electrode’s microstructure is homogenized, and transport properties are described using effective parameters. This scale treats the electrode as a continuous medium using averaged properties like effective ionic and electronic conductivity to predict overall battery performance. The macroscale approach simplifies the complexity of the microstructure into usable parameters for large-scale simulations, aiding in the design and optimization of PTMA-based batteries.

In this contribution, we focus on the mesoscale because it is crucial for the behavior at the macroscopic scale, where most experimental measurements are performed to investigate battery performance under different operating conditions. One such critical observation is the battery’s capacity, which does not solely depend on the theoretical capacity derived from the intrinsic material properties at the molecular level. Instead, it is also strongly influenced by the electrode’s microstructure at the mesoscale. Note that the electrolyte in the porous electrodes serves as a reservoir for the ions required for the redox-reaction that ensures charge neutrality. As far as electronic transport is concerned, a minimum amount of electronically conductive additives must be ensured to provide complete percolation through the entire electrode thickness to guarantee the activation of all active material particles. Once this amount is reached, no further conductive additives should be added to achieve an optimum specific capacity. More precisely, the percolation threshold is at 8 wt % SuperP according to (23), where polymer-based electrodes with varying fractions of SuperP are investigated via conductivity impedance measurements. In particular, it has been shown that there is no electrochemical activity below this percolation threshold, while a range of capacity utilization values can be achieved above the percolation threshold. This can be interpreted as follows: A minimum percolation path is required to ensure proper contact within the electronic transport network and to trigger electrochemical activities, but the amount of conducting additives is not sufficient to activate all PTMA particles. By increasing the amount of SuperP, the maximum achievable absolute capacity is increased until a saturation value is reached, determined by the amount of PTMA. For the purpose of investigating quantitative structure–property relationships, three different electrode compositions with a content between 30 and 60 wt % of SuperP have been chosen to ensure a well-percolated electrode that also exhibits a high capacity utilization as shown in (23).

The aim of this study is to investigate the connection between the mesoscopic and macroscopic descriptions of transport phenomena in porous electrodes, specifically focusing on how the morphological characteristics of the electrodes influence effective transport properties. To make this relationship explicit, analytical formulas are derived that express the effective transport parameters in terms of geometrical descriptors. To validate these formulas, we compare their predictions with results from direct simulations performed on the microstructures.

The rest of the paper is organized as follows. Section 2 explains materials and methods considered in this contribution, including a description of the manufacturing procedure of the three polymer-based electrodes with PTMA as active material (Section 2.1), and the subsequent imaging via 3D FIB-SEM tomography (Section 2.2). Then, in Section 2.3, the segmentation of the 3D image data into active material, carbon-binder domain and pore space is described. Afterward, in Section 2.4, various geometrical descriptors are explained which are used to characterize the 3D morphology of the electrodes, whereas Section 2.5 contains a description of spatially resolved numerical simulations of effective transport properties that are closely related to the electrochemical performance of the electrodes. Section 3 contains the results which have been obtained in the present paper. In particular, in Section 3.1, the differences between the nanostructures of the three electrode samples are statistically analyzed. Next, in Section 3.3, relationships between the 3D nanostructure of the polymer-based battery electrodes and effective transport properties are investigated by means of analytical regression formulas. Finally, Section 4 concludes and provides an outlook on possible future research.

## 2 Materials and Methods

### 2.1 Materials Synthesis and Electrode Manufacturing

The electrodes considered in the present paper contain SuperP (specifically Super P Conductive, 99+%, metals basis, manufactured by Alfa Aesar, USA) as the conductive additive. Carboxymethyl cellulose (CMC, Sigma-Aldrich, USA) is used as the binder. Both SuperP and CMC were used as received, without any additional purification. The active material, PTMA, was synthesized via emulsion polymerization following the procedure described in (34). In this study the particle type P2 from (34) is considered, consisting of nanoparticles with a mean particle size of 73 nm.

Furthermore, the manufacturing process of the polymer-based electrodes considered in this study is described in detail in (34). For convenience, a brief summary of the main processing steps is provided here. In particular, we consider three electrodes that differ in their material composition. The different ratios of PTMA, SuperP and CMC are listed in Table 1, where the sample name corresponds to the weight percentage of SuperP. For each sample, 500 mg of active material were dispersed in 5 mL water using a Zentrimix 380R disperser (Andreas Hettich GmbH & Co, Germany) at 1,500 rpm for 1 h. The electrode films were manufactured using a BYK byko-drive XL doctor-blading setup, where the slurry was applied to KOH-etched aluminum foil with a blade gap set to 200 μm and a casting speed of 250 mm s^–1^. The coated films were annealed for 18 h at 80 ^◦^C under ambient atmosphere. The resulting electrodes had thicknesses ranging from 70 to 150 μm, depending on the slurry viscosity, and are referred to as SP30, SP45, and SP60. The electrodes were then punched into 15 mm diameter discs using an MTI Corporation crimper, with a final electrode area of 1.76 cm^2^ and a loading of 1 to 3 mg after drying.

**Table 1:** Material Composition of Three Organic Radical Battery Electrodes with Different Amounts of Active Material PTMA and Conductive Additive SuperP, Where the Weight Percentage of the CMC Binder Is Kept Constant

Material Sample	SP30	SP45	SP60
PTMA/*wt*. – %	65	50	35
SuperP/*wt*. – %	30	45	60
CMC/*wt*. – %	5	5	5

### 2.2 Tomographic Imaging

In this section, we describe the sample preparation and imaging of the samples via 3D FIB-SEM tomography.^35,36^ Each of the three polymer electrodes was first cut into 1 × 3 mm sections with a scalpel and fixed to a standard aluminum SEM holder using a carbon adhesive pad. They were then sputtered with a layer of gold approximately 10 nm thick to further improve the electronic conductivity at the sample surface. Infiltration with fillers such as resins or silicone was avoided. On the one hand, this was due to the concern that infiltration would significantly alter the structures to be measured. On the other hand, the infiltration of polymers creates a contrast problem, which in turn makes it difficult to distinguish between the sample and the infiltration material. Finally, without infiltration it is possible to increase the speed of cutting the sample with the focus ion beam, which results in a shorter measurement time. The samples were then transferred to the FIB-SEM, a ZEISS Crossbeam 340 at the Centre for Correlative Microscopy and Spectroscopy (CCMS). The crossbeam has a Gemini I electron column that was operated at a low voltage of 1 keV for the tomography imaging measurements. The low voltage was chosen to minimize the penetration depth of the primary electrons, which avoids the occurrence of artifacts. Moreover, the low voltage simplifies the segmentation of the resulting image data since the shallow depth of field means that areas that are not in the slice plane quickly become blurred. The gallium ion gun of the crossbeam, mounted at an angle of 54° to the electron column, was operated at an acceleration voltage of 30 keV. To obtain a good view of the area of the sample that is to be imaged, an area of approximately 40 μm × 40 μm was first removed from the sample using a gallium current of 30 nA. For sequential image acquisition, both the secondary electron in-lens detector integrated in the electron column and the detector built into the side of the microscope chamber, which also detects secondary electrons, were used. After polishing the side of the previously exposed area intended for tomography with a gallium current of 700 pA and setting the tilt compensation and dynamic focus, the serial sectioning process of the tomography was started. The cutting depth and pixel size were always chosen to be the same. More precisely, a pixel size of 15 nm is used in case of the samples SP30 and SP45. Due to a new gallium source, allowing for a longer measurement, a pixel size of 10 nm was used for the sample SP60. The sizes in numbers of voxels in each spatial direction of the three reconstructed samples are reported in Table 2.

**Table 2:** Sample Size in *x*- , *y*-, and *z*-Direction, Respectively^a^

Sample	*x* [num. voxels]	*y* [num. voxels]	*z* [num. voxels]
SP30	1237	700	1891
SP45	1796	700	880
SP60	2924	2122	1397

a Note that the *y*-direction corresponds to the direction from the current collector to the separator, which is the main direction of ionic and electronic transport.

### 2.3 Image Processing

In the following, we describe the process of segmenting the raw FIB-SEM image data into three components: active material (PTMA), carbon-binder domain (CBD), which consists of SuperP and CMC, and pore space. After completing tomographic imaging described in Section 2.2, the raw data was prepared for classification using the software Fiji.^37^ First, the SIFT-based image drift correction was applied.^38^ The data was then denoised by applying a 2D nonlocal means filter.^39^ Due to the noninfiltrated nature of the measurement, the shine through artifacts, which represent the background of the sample in areas with no material in the cutting plane, had to be detected and removed. A U-net based 3D neural network was used for this challenging postprocessing step.^40^ The network was trained on many similar previous measurements not directly related to the present paper, which were classified using a random forest approach.^41^ The removal of shine through artifacts using neural networks worked well for the samples SP45 and SP60. However, the sample SP30 showed many charging artifacts that the network had not been previously trained on, causing the network to fail. Therefore, the previously mentioned random forest approach was used. Here, both input channels, the InLens detector signal and the angled chamber detector signal, were used to manually train a new random forest classifier using the ilastik software package.^42^ Although much slower than the neural network approach, this ultimately resulted in a satisfactory removal of the shine through artifacts.

**Figure 1:**
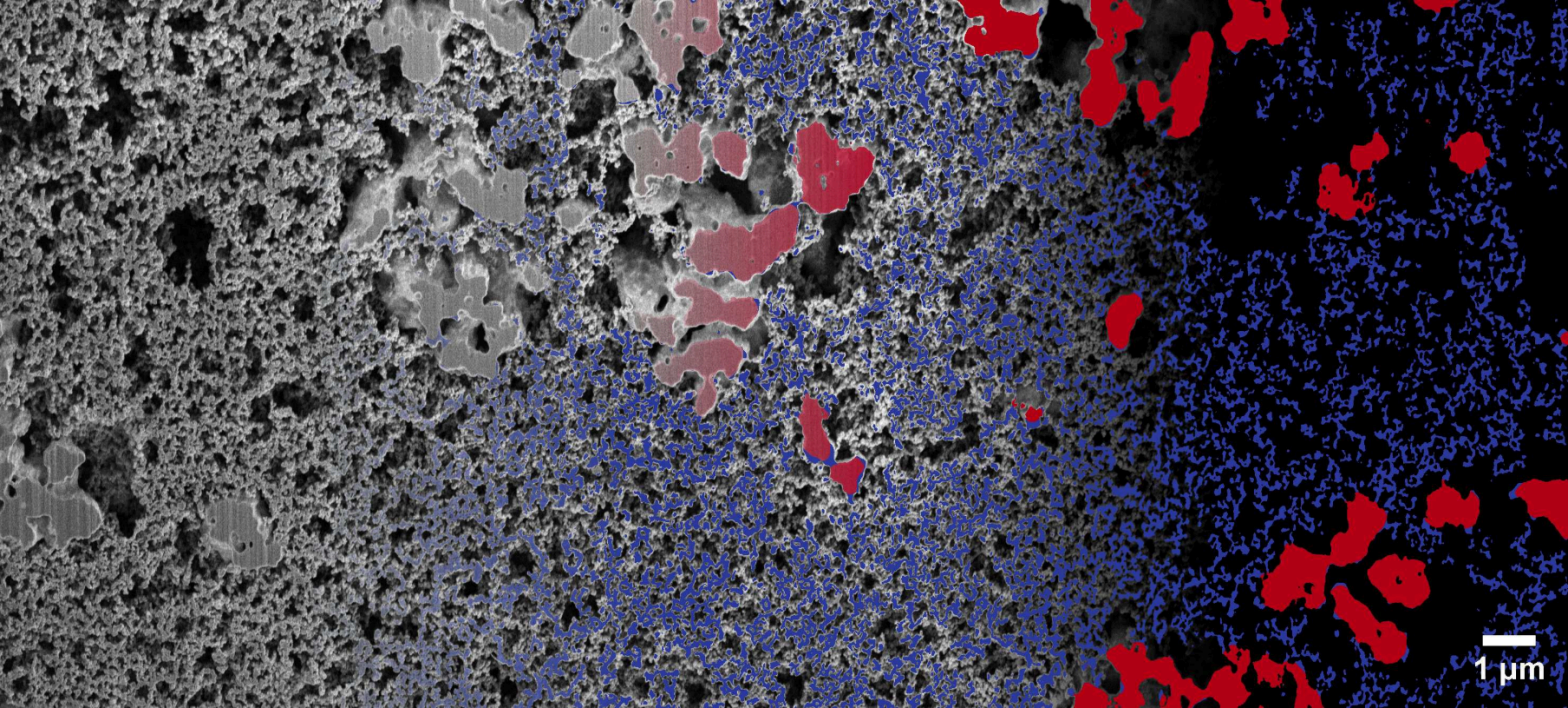
Exemplary selected 2D slice of sample SP60. From left to right, a continuous transition is shown from the grayscale SEM image to the segmentation into active material (red), carbon-binder domain (blue), and pore space (black).

**Figure 2:**
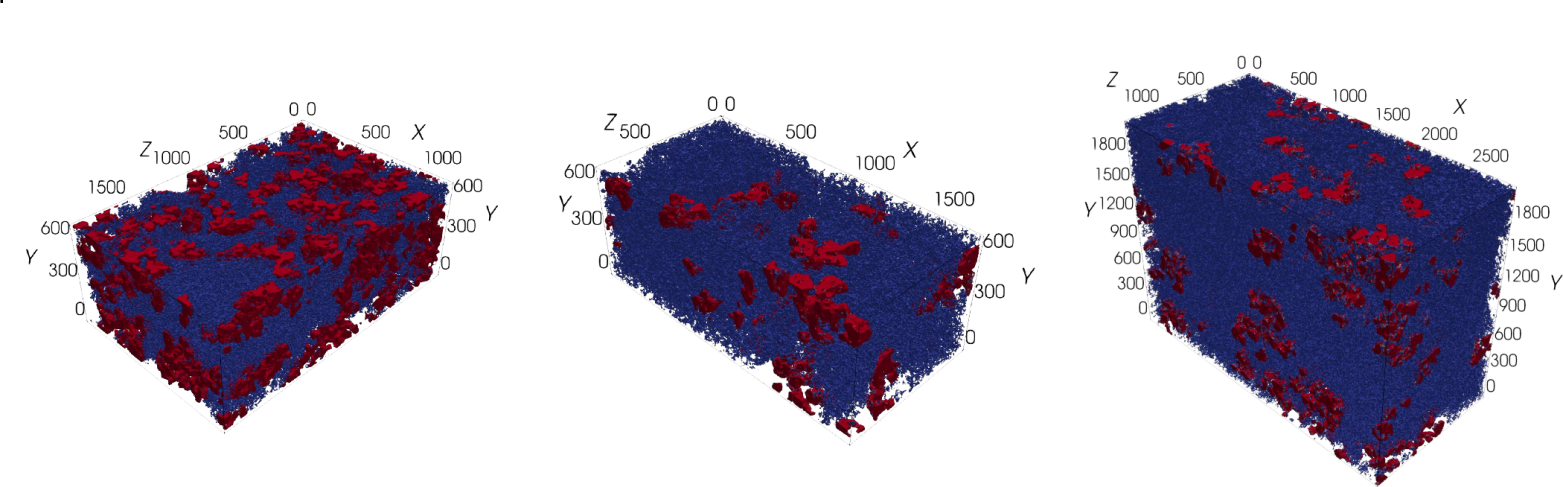
Visualization of the segmented 3D images. The PTMA phase is shown in red, the carbon-binder domain is shown in blue, and the pore space is kept transparent. The axis scale is in voxel, where the samples on the left (SP30) and in the middle (SP45) have a voxel size of 15 nm. The sample displayed on the right (SP60) has a voxel size of 10 nm.

The resulting binary image was further classified into the active PTMA phase and the carbon-binder domain. For this purpose, a morphological approach was used that exploits the fact that the carbon-binder domain consists of much smaller, clustered particles compared to the PTMA phase. Based on this knowledge, a local thickness filter that is included in the Fiji software package was applied.^37^ The result was then thresholded into the two remaining phases.^43^ The segmentation procedure and the resulting segmented 3D images are visualized in Figures 1 and 2, respectively.

### 2.4 Geometrical Descriptors

The following section provides a brief explanation of several geometrical descriptors used to characterize the 3D morphology of the polymer-based battery electrodes. Superscripts are used to indicate the specific phase for which each descriptor is calculated. Specifically, the superscripts AM (active material), CBD (carbon-binder domain) and P (pore space) are used to refer to the PTMA phase, the carbon-binder domain, which includes both SuperP and CMC, and the pore space, respectively. Furthermore, the superscript is omitted in case when the underlying phase is clear from the context.

#### 2.4.1 Volume Fraction

One of the primary geometrical descriptors is the volume fraction ε ∈ [0, 1] of the phase under consideration. This quantity is estimated from 3D image data using the well-known point-count method.^44^ Besides the globally computed volume fraction ε, the heterogeneity of the electrode nanostructures will be quantified by computing the volume fraction for nonoverlapping cutouts of size 100 μm × 100 μm × 100 μm.

#### 2.4.2 Specific Surface Area

In addition to volume fraction, we consider the specific surface area, which will be denoted by *S*. It is defined as the surface area between the considered phase and its complement divided by the volume of the sampling window. This quantity is estimated from voxelized 3D image data using an approach presented in (45), which is based on a convolution of the image with a 2 × 2 × 2 kernel. Moreover, the specific surface areas of the different phases will be used to define more sophisticated geometrical descriptors as described in the next paragraph.

#### 2.4.3 Interfaces

In addition to the specific surface areas of the three phases, we consider the specific area of the interface between active material and the carbon-binder domain, denoted by *S*^AM∩CBD^, which is of importance with regard to electronic transport. Analogously, the specific area of the interface between active material and the pore space, denoted by *S*^AM∩*P*^, is considered since a sufficiently large interface is required to ensure an ionic flux into the active material.

#### 2.4.4 Mean Chord Length

A further geometrical descriptor is the chord length distribution,^46,47^ where a chord is a line segment that is completely contained in a predefined phase and can not be extended further without intersecting the complementary phase. In general, the probability distribution of chord lengths depends on the orientation of the line segments. We compute the chord length distribution for the three Cartesian axes directions. In particular, for each of these three directions, we compute the mean value of the corresponding chord length distribution. In the following, the average of these three mean values, denoted by μ(*C*), is used.

#### 2.4.5 Constrictivity

In order to explain the notion of constrictivity, we first recall the concepts of the continuous phase size distribution (CPSD) and simulated mercury intrusion porosimetry (SMIP). Namely, CPSD:[0,*∞*) → [0,1] is a function, where the value CPSD(*r*) is given by the volume fraction of the phase under consideration, which can be covered by (possibly overlapping) spheres with radius *r* ≥ 0 such that the spheres are completely contained in the considered phase.^47,48^ Furthermore, by *r*_*max*_ the maximum radius *r* > 0 is denoted such that CPSD(*r*) ≥ ε/2 where ε is the volume fraction of the considered phase. The concept of SMIP is similar to that of CPSD, with the only difference that the value SMIP(*r*) of SMIP:[0,*∞*) → [0,1] is given by the volume fraction of the phase under consideration, which can be covered by (potentially overlapping) spheres with radius *r* forming an intrusion from a predefined direction. Analogously to *r*_*max*_, by *r*_*min*_ the maximum radius *r* > 0 is denoted such that SMIP(*r*) ≥ ε/2. In general, *r*_*min*_ depends on the direction of the intrusion. Thus, *r*_*min*_ is computed for each of the three axes directions separately and, subsequently, the average of the three obtained values is used. The constrictivity β of the phase under consideration is then defined as  It is a measure for the strength of bottleneck effects and has been originally introduced in (49). Since *r*_*min*_ ≤ *r*_*max*_ by definition, it holds that β ∈ [0, 1], where β = 1 corresponds to the situation that there are no constrictions within the considered phase. In (50−52) it has been shown that the constrictivity β of the pore space has a significant impact on effective macroscopic properties of porous media such as effective diffusivity or permeability.

#### 2.4.6 Mean Value and Standard Deviation of Geodesic Tortuosity

A further transport-relevant geometrical descriptor is the so-called geodesic tortuosity. Besides geodesic tortuosity, there exist several other concepts of tortuosity in the literature, see e.g., (24) and (53) for a comprehensive overview. According to the nomenclature proposed in (24), the geodesic tortuosity considered in the present paper corresponds to τ_dir_geodesic_. It will be denoted by τ_g_ in the following. This purely geometrical descriptor captures the windedness of transport paths, which are completely contained in a predefined phase. From 3D image data, the distribution of τ_g_ is determined by computing the lengths of shortest paths from randomly selected voxels within the considered phase, which belong to a predefined starting plane, to a parallel target plane, divided by the distance between those two planes, where shortest paths are computed using Dijkstra’s algorithm.^54^ Usually, the starting and target planes are chosen orthogonal to the relevant transport direction. For the image data considered in the present paper, we compute the distribution of τ_g_ with respect to each of the three Cartesian axes directions. The mean value μ(τ_g_) of τ_g_ is then determined by averaging over all shortest path lengths divided by the distance between the starting and target planes. Furthermore, the empirical standard deviation σ(τ_g_) of these normalized path lengths is considered.

#### 2.4.7 Local Variants of Geometrical Descriptors

The computation of the geometrical descriptors stated above allows us to capture “global” morphological features of the electrodes. However, in case of lithium-ion batteries, it is well-known that local heterogeneity of electrodes has a strong influence on the resulting electrochemical performance.^55−58^ This is also expected to hold for polymer-based batteries, which motivates the computation of local variants of geometrical descriptors. For this purpose, the sampling window of each of the three material samples SP30, SP45 and SP60 is partitioned into nonoverlapping cutouts of size 1 μm × 1 μm × 1 μm. Next, the geometrical descriptors considered in the present paper are computed separately on each of these cutouts, which results in probability distributions of these local descriptors. In particular, Section 3.1 contains results regarding the distribution of local volume fraction as well as of the local specific surface area of interfaces.

### 2.5 Numerical Simulation of Effective Transport Properties

As discussed in the Introduction, the primary objective of this study is to explore how electrode morphology affects the effective transport coefficients. To establish a quantitative link between microstructural features and transport behavior, we derive analytical expressions that relate the effective transport properties—such as ionic and electronic conductivity—to specific geometrical descriptors of the electrode as listed in the previous section.

The intrinsic transport coefficient *D* reflects the ideal transport properties of a uniform material, while the effective transport coefficient *D*_eff_ incorporates the effects of obstacles and phase connectivity. The latter is a scaled version of the intrinsic coefficient that accounts for the complexity of the 3D porous microstructure and is crucial for macroscopic or multiscale simulations. It incorporates the influence of the real microstructure geometry, enabling more accurate predictions of transport phenomena.

To quantify the influence of morphology on transport processes, we use the M-factor, which is defined as the ratio between the effective transport coefficient and the intrinsic transport coefficient:
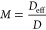
To quantify this relationship, we derive analytical formulas to represent the M-factor as a function of key geometrical descriptors. To validate these formulas, we compare the analytically derived M-factor with one derived by direct simulations on the 3D microstructures. The notion of the M-factor is closely related to the effective tortuosity^59−62^ τ_eff_, which is defined in the literature as
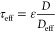
where ε represents the volume fraction of the phase of interest (e.g., the solid phase for electronic conduction or the pore space for ionic diffusion). Thus, it holds 

For a general simulation of transport processes, the effective tortuosity must be computed in all three spatial directions: *x*, *y*, and *z*. However, the primary transport direction in batteries is usually orthogonal to the current collector. Therefore, in this study, we focus solely on the effective tortuosity in the main transport direction. Depending on the homogeneity of the electrodes and the scale of the simulation (e.g., in full-cell simulations), the effective tortuosity in the other directions may be important, but here we limit our characterization of effective transport coefficients to the primary transport direction, which is along the *y*-axis as indicated in Figure 2.

The effective tortuosity in this main direction is computed via finite element simulations by solving the steady-state diffusion equation within the 3D microstructure:
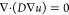
where *u* represents the concentration or potential field, and *D* is the intrinsic transport coefficient. Dirichlet boundary conditions are applied along the main transport direction, while no-flux boundary conditions are used on the remaining boundaries. The flux from this simulation is compared to the flux in a corresponding homogenized domain with no microstructural features, allowing us to compute the effective transport coefficient *D*_eff_. Further details on the computation of τ_eff_ are provided in (62).

**Figure 3:**
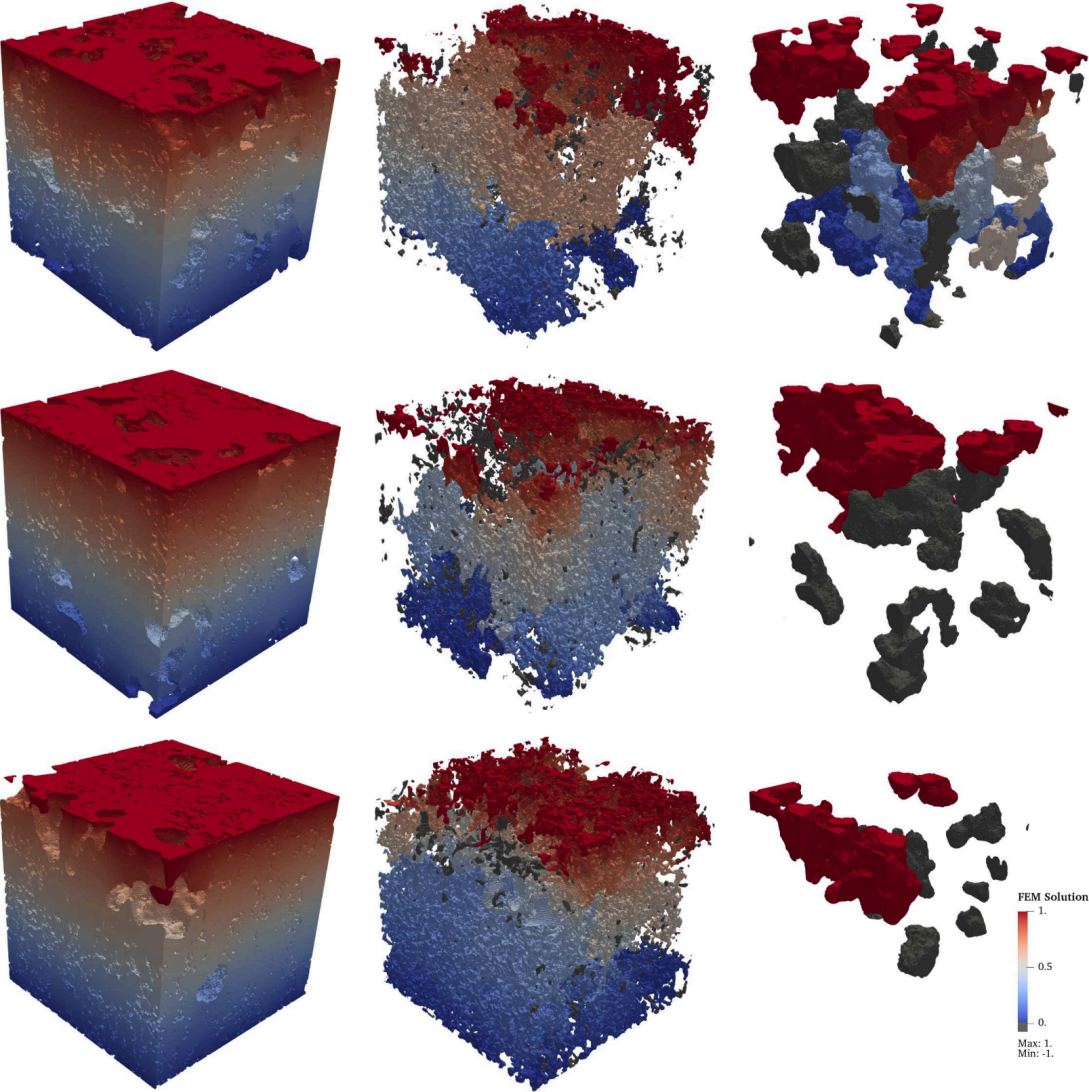
Visualization of the finite element solution of the transport problem to compute the effective transport coefficients in the three phases (PTMA, CBD, pores), where the transport direction is from top to bottom, i.e., in the main direction of transport. The common color scale is shown at the right-hand side of the third row of images, where the red color corresponds to the value 1, and the blue color to 0. The gray color is used to indicate all parts that are neither connected to the top nor to the bottom surface and, therefore, do not contribute to the transport process. The electrolyte concentration in the pore space is shown in the left column, the electric potential distribution within the carbon-binder domain in the middle column, and the ionic concentration within PTMA in the right column. Note that the different rows correspond to the subsamples SP30.1, SP45.2, and SP60.2 (from top to bottom). In particular, the PTMA phase is not connected from top to bottom, i.e., is not percolating, in the case of subsamples SP45.2 and SP60.2.

The imposed boundary conditions create a gradient in the solution along the main direction of electronic and ionic transport, as shown in Figure 3, where the transport is predominantly from top to bottom. The ionic transport through the electrolyte is shown in the left column of the figure, while the center column illustrates the electronic transport pathways within the SuperP phase. The right-hand column displays transport within the PTMA phase, which does not form a fully percolating network. However, full percolation of PTMA is not required, as ions are delivered to the PTMA particles through the surrounding electrolyte, rather than through direct transport from one PTMA particle to another. The simulations also show localized 3D effects that illustrate the influence of the microstructure on the transport process.

Given the total dimensions of the samples SP30, SP45 and SP60, see Table 2, subsamples have been considered of size not larger than 1024 × 1024 × 1024 voxels, i.e., a box with a maximum length of 1024 along each axis. A list of all subsamples is given in Table 3. The FEM simulations have been performed on each of these subsamples. These computations have been performed on the HPC-cluster HSUper (see Acknowledgments) by a finite element code based on the deal.II library.^63^

**Table 3:**
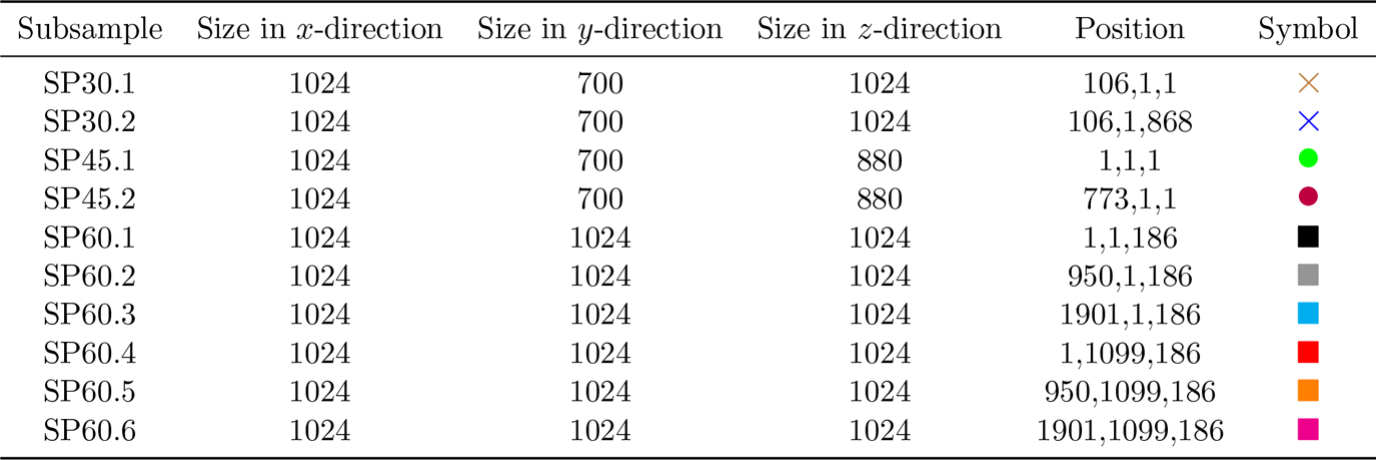
Information Regarding the Subsamples Containing (from Left to Right): Name of the Subsample, Size of the Subsample in *x*-, *y*-, and *z*-Direction (in Number of Voxels), the Position of the Lower Left Voxel of the Subsample with Respect to the Corresponding Sample, and a Unique Symbol as Visual Identifier

## 3 Results and Discussion

### 3.1 Statistical Analysis of Nanostructure via Subsamples

**Figure 4:**
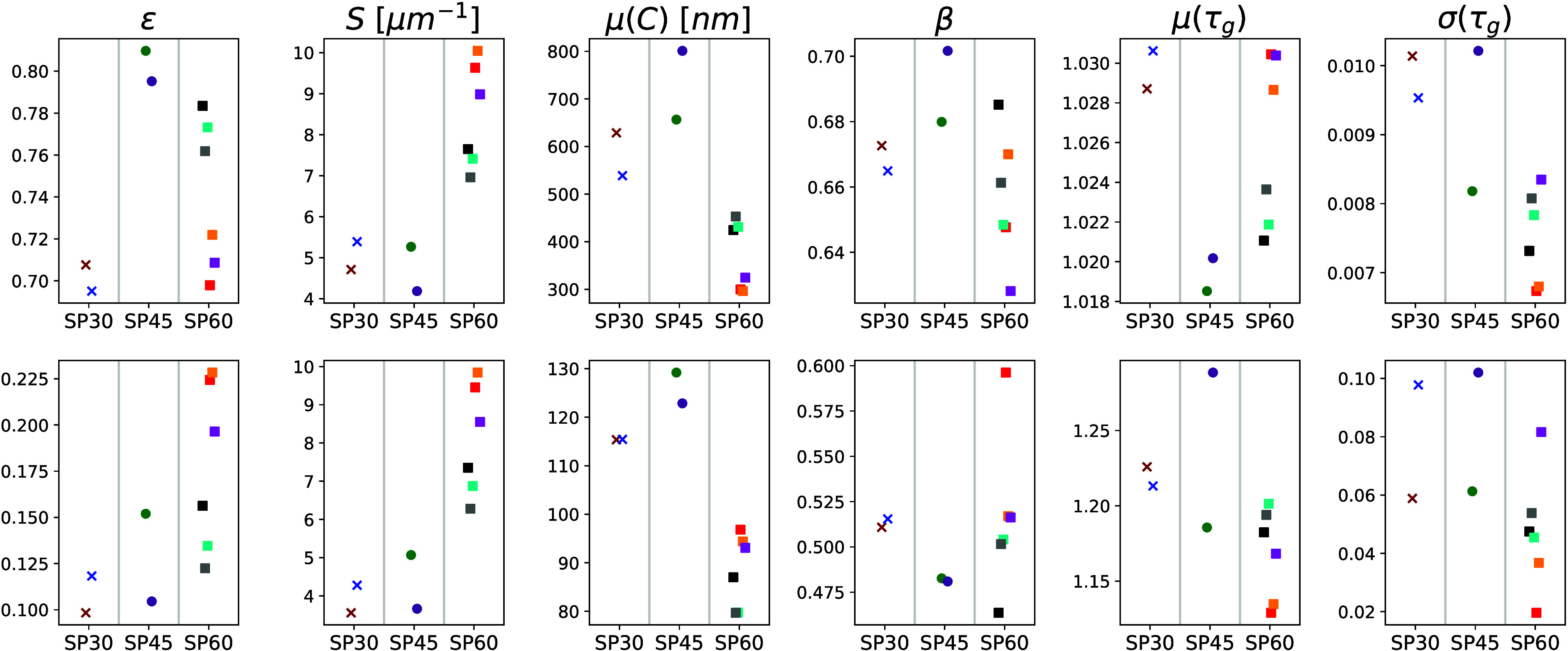
Morphological characterization of subsamples of the polymer-based electrodes SP30, SP45, and SP60. The columns correspond to the geometrical descriptors stated in Section 2.4. The results shown in the upper row concern the pore space, whereas the lower row refers to the CBD. The shapes and colors relate to the sample and subsample symbols as given in Table 3, respectively.

To quantitatively compare the electrode samples SP30, SP45 and SP60, the geometrical descriptors detailed in Section 2.4 are computed for all subsamples (see Table 3 for further information regarding the subsamples). The results are shown in Figure 4. In particular, the volume fractions ε^P^ and ε^CBD^ of pore space and the carbon-binder domain lie in the ranges of [0.70, 0.81] and [0.10, 0.23], respectively. Notably, there is significant variability in porosity within the subsamples of SP60. This variability is even more pronounced with respect to the volume fraction of the carbon-binder domain. A similar behavior is also observed with regard to other geometrical descriptors and the M-factor.

Furthermore, the specific surface area of the pore space (*S*_P_) as well as the carbon-binder domain (*S*_CBD_) is significantly higher for the sample SP60 compared to the other two samples as shown in the second column of Figure 4. This finding is unexpected, as there is no significant difference in specific surface area when comparing SP30 and SP45 despite the fact that the weight percentage of SuperP differs between SP30 and SP45 by the same amount as between SP45 and SP60.

The mean chord length (μ(*C*)), as depicted in the third column of Figure 4, exhibits a strong correlation with the volume fraction of the respective phase, with correlation coefficients of 0.77 for the pore space and 0.96 for the carbon-binder domain. Moreover, similar to the specific surface area, this characteristic also indicates a finer structure of both the pore space and the CBD for SP60 compared to SP30 and SP45.

With regard to the carbon-binder domain, there is a high variation of constrictivity within the considered subsamples only in case of sample SP60, see Figure 4. Compared to the mean chord length, the constrictivity of the respective phases is less correlated with the volume fraction. More precisely, the values of these two descriptors have a correlation coefficient of 0.58 for the pore phase and 0.55 in case of the CBD. Therefore, a multiparameter formula for predicting the M-factor in Section 3.3 that incorporates both ε and β is considered more promising than a formula based solely on ε.

Regarding the mean geodesic tortuosity of the pore space, no significant difference are observed between the samples, as indicated by the small range of values in the corresponding plots shown in Figure 4. This is likely due to the high porosity values, which typically ensure almost straight transport paths for ions. However, the situation is different for the CBD. With volume fractions of CBD ranging between 10% and 23%, the normalized lengths of shortest transport paths within the CBD are significantly larger than one, with variations both between the three samples and within individual subsamples. The sample with the largest variation between the corresponding subsamples is SP45. Besides mean geodesic tortuosity, the standard deviation of geodesic tortuosity is also considered as one possible descriptor that quantifies the local heterogeneity of the nanostructure. For example, the two subsamples of SP30 have a similar value of μ(τ_g_), but show a clear difference in σ(τ_g_). Moreover, the low correlation between σ(τ_g_) and μ(τ_g_) as well as ε indicates that a combination of these three quantities is well suited for predicting the M-factor, see eq 4 below.

### 3.2 Analysis of Local Heterogeneity

**Figure 5:**
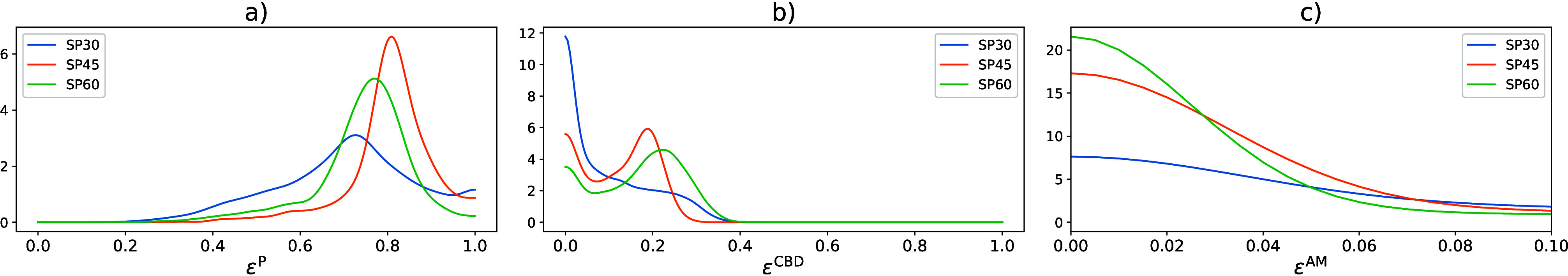
Distribution of local volume fraction of pore space (a), CBD (b), and active material (c).

Besides the computation of geometrical descriptors on each subsample, we also consider the distribution of local volume fraction for each of the three phases (PTMA, CBD, pores) to quantify the local heterogeneity of electrodes. For this purpose, nonoverlapping cutouts with a size of 1 μm × 1 μm × 1 μm are used as described in Section 2.4. The results obtained for the distribution of local volume fraction are shown in Figure 5. In particular, the three electrodes (SP30, SP45, SP60) exhibit clearly different distributions of local porosity not only in the mean porosity—expected due to the different material compositions—but also in the shape of the distribution. For example, in the case of SP30 a significant amount of cutouts exhibit a porosity between 50% and 60%, whereas SP45 primarily consists of regions with porosity values larger than 70%. The center plot of Figure 5 shows that for all three samples, several cutouts contain no CBD at all, which is most pronounced in SP30. Furthermore, the spatial distribution of the active material is far from homogeneous, indicated by the plot on the right-hand side of Figure 5. More precisely, the vast majority of cutouts in SP45 and SP60 lack any active material, while those containing it can exhibit volume fractions up to 100%. This is due to the nanoparticles used for synthesizing the active material via emulsion polymerization, which tend to agglomerate to large clusters, resulting in local PTMA volume fractions close to 100%.^34^

For SP30, the amount of cutouts without active material is significantly lower. More precisely, the fraction of cutouts that do not contain any active material is 13% for SP30 compared to 56% for SP45 and 52% for SP60.

**Figure 6:**
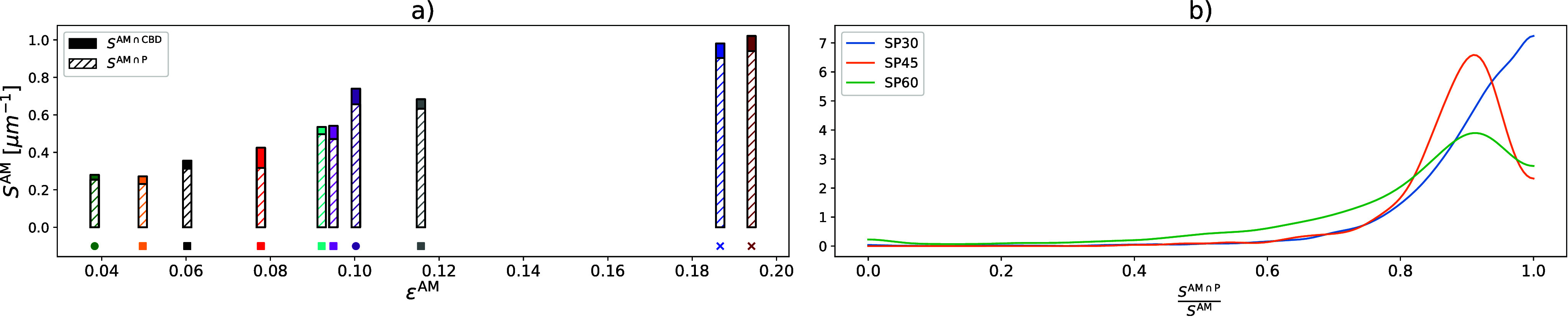
(a) Specific surface area of active material including the contribution of the interface between PTMA and the CBD (filled part) and the interface between PTMA and the pore space (shaded part). (b) Distribution of the local ratio of the specific interface area between active material and pore space divided by the (entire) specific surface area of active material.

The interface area between PTMA and the other two phases is crucial for the electrochemical performance, as shown in the left-hand plot of Figure 6. Notably, the majority of the active material surface is in contact with the pore space. In particular, SP30 has the largest interface area between active material and the CBD, even though SP30 is the sample that contains the least amount of SuperP. Moreover, the right-hand plot of Figure 6 shows the distribution of the (local) ratio of the specific interface area between active material and pore space (*S*^AM∩*P*^) relative to the (entire) specific surface area of active material (*S*^AM^). This ratio is computed for each nonoverlapping cutout with a size of 1 μm × 1 μm × 1 μm to investigate the spatial distribution of interfaces. Almost all local volumes (except for SP30) show some degree of contact between the active material phase and the CBD, a crucial property for the proper electrode function. Moreover, sample SP60 exhibits a non-negligible amount of regions, where the interface between pore space and active material only makes up for 40 to 70% of the (entire) specific surface area of active material, which in turn results in a comparatively large contact area between CBD and active material.

**Figure 7:**
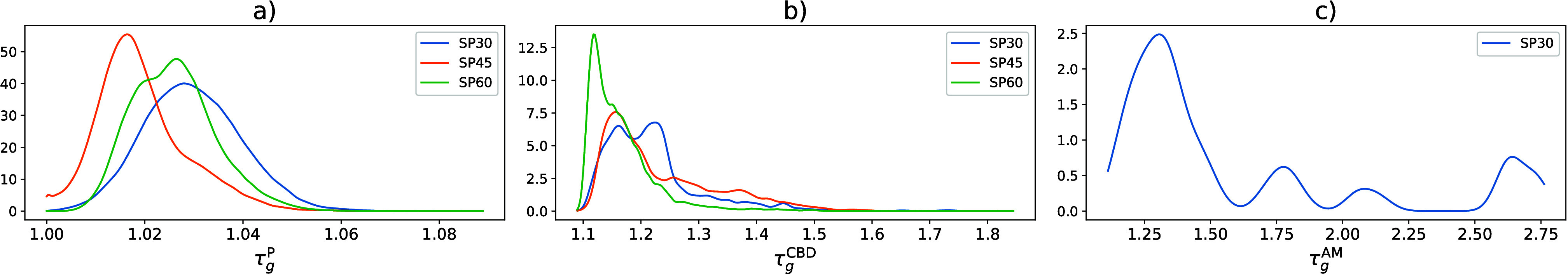
Distribution of local geodesic tortuosity of pore space (a), CBD (b), and active material (c). Note that in the case of the active material phase, there are no shortest paths from the starting plane to the opposite plane in the case of SP45 and SP60.

Finally, we consider the geodesic tortuosity of the three electrode phases in more detail, see Figure 7 which depicts the distribution of local geodesic tortuosity of the different phases and samples. In particular, it can be observed that there is no path within the active material phase between the two opposing planes in case of samples SP45 and SP60, which is not necessarily required for a proper functioning of the electrode. Furthermore, in terms of pore space and CBD, SP30 and SP60 exhibit a high degree of similarity. However, with regard to SP45, the shortest paths in the pore space tend to be slightly shorter, while in the CBD, they tend to be slightly longer compared to the other samples.

### 3.3 Microstructure–Property Relationships

In addition to the computation of geometrical descriptors, FEM simulations have been performed, as stated in Section 2.5, in order to determine the effective tortuosity and the M-factor of the CBD and pore space for all subsamples considered in the present paper, see Table 4.

**Table 4:**
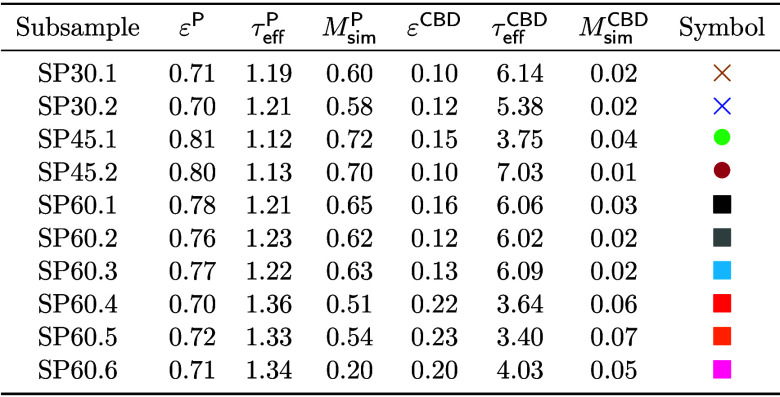
Effective Tortuosity and M-Factor of Pore Space and CBD Obtained by FEM Simulations, Together with the Volume Fraction of the Corresponding Phase

We now investigate the relationship between various geometrical descriptors and the M-factor of the CBD and the pore space, respectively. Many formulas have been proposed in literature for predicting the M-factor of various materials and different transport modes from geometrical descriptors.^52,64,65^ In the present paper, we discuss four of these formulas, which have demonstrated significant prediction accuracy for both the pore space and CBD, as quantified by the deviation measures MAPE and *R*^2^. Recall that the mean absolute percentage error (MAPE) is given by
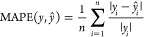
where  is an *n*-dimensional vector of predictions of some “ground truth” data *y* = (*y*_1_, ..., *y*_*n*_). A further deviation measure is the coefficient of determination (*R*^2^) which is defined as
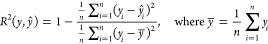


The first formula for predicting the M-factor relies solely on the volume fraction ε of the respective phase, i.e., we consider the predictor  given by
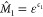
1where *c*_1_ > 1 is some constant. Various numerical values have been proposed in literature for *c*_1_, ranging from 1.5 (Marshall formula) to 2 (Buckingham formula).^66−68^ For the data considered in this study, the value *c*_1_ = 1.71 as proposed in (64) provided the best results in terms of both MAPE and *R*^2^, and will be used in the following analysis.

The second formula provides the predictor  which not only accounts for the volume fraction ε of the phase, but also incorporates the mean geodesic tortuosity μ(τ_g_) and constrictivity β. This captures both geometric bottleneck effects and the relative path lengths. The formula is expressed as
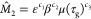
2where *c*_1_ > 0, *c*_2_, *c*_3_ ≥ 0 are some constants. The formula in eq 2 was originally proposed in^65^ with the constants (*c*_1_, *c*_2_, *c*_3_) = (1.15, 0.37, – 4.39). However, the vector of constants (*c*_1_, *c*_2_, *c*_3_) = (1, 0, – 8.45), proposed later in (50), proved to be more effective for predicting the M-factor of the data considered in the this paper and will therefore be used in the subsequent analysis.

A modified version of the formula given in eq 2 can be found in (52). It provides the predictor  of the M-factor, where the constrictivity β appears in the exponent of the volume fraction ε as follows:
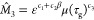
3with some constants *c*_1_, *c*_2_, *c*_3_ such that *c*_1_ + *c*_2_ ≥ 0, *c*_3_ ≤ 0. In the following we consider the numerical values (*c*_1_, *c*_2_, *c*_3_) = (1.25, – 1.25, – 7.82) which have been proposed in (50).

Finally, a formula is considered which has been introduced in (69) and provides a further predictor (denoted by  for the M-factor. It assesses the impact of the pore structure on the M-factor by considering the standard deviation of the geodesic tortuosity σ(τ_g_) instead of the constrictivity β. More precisely, the predictor  is given by
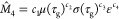
4where the choice (*c*_1_, *c*_2_, *c*_3_, *c*_4_) = (1.18, – 9.17, 0.03, 1.02) of the constants *c*_1_, *c*_2_, *c*_3_, *c*_4_ proposed in (50) yields an improved fit of the M-factor for the data considered in the present paper, compared to the numerical values of *c*_1_, *c*_2_, *c*_3_, *c*_4_ derived in.^69^

### 3.4 Evaluation of Prediction Power

**Figure 8:**
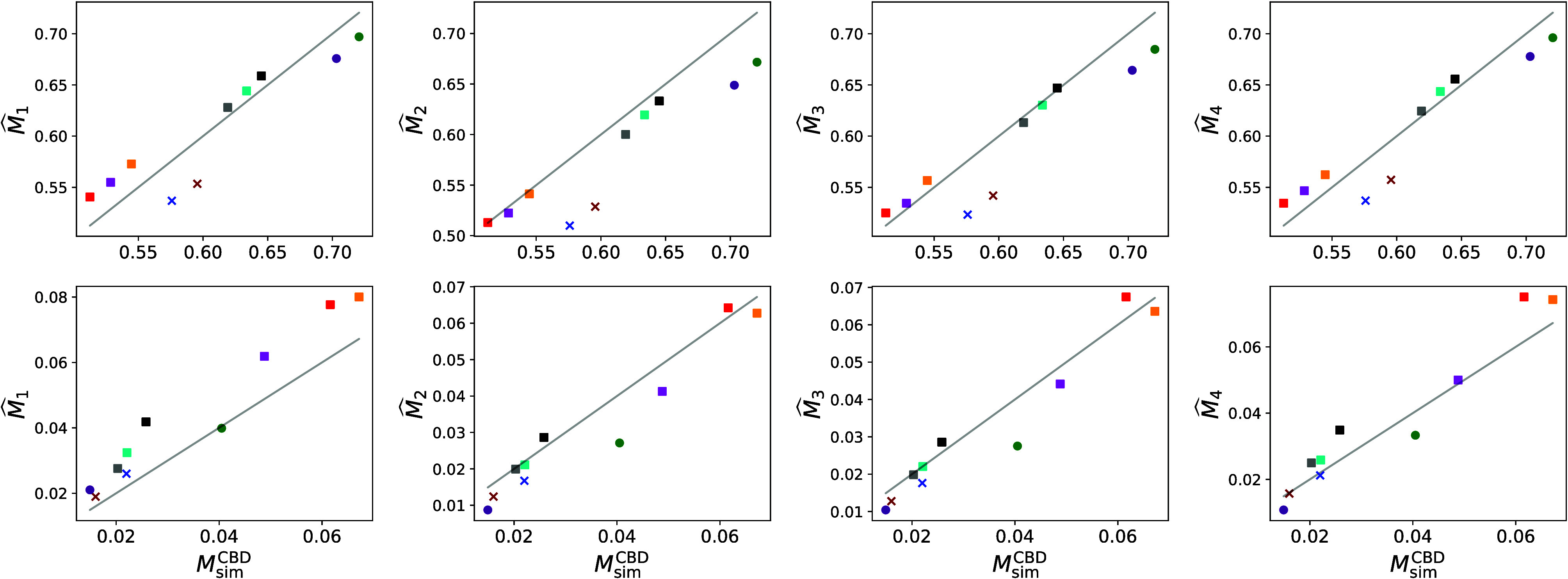
Prediction of M-factor via analytical regression formulas. The upper row contains the results which have been obtained for the pore space, the lower one those for the CBD. The shape and color codings refer to the individual subsamples as stated in Table 3.

Using the formulas given in eqs 1, 2, 3, and 4 for predicting the M-factor, using the constants *c*_1_, *c*_2_, *c*_3_, *c*_4_ as specified above, leads to the results shown in Figure 8.

A quantitative evaluation of the results shown in Figure 8 in terms of MAPE and *R*^2^ is given in Table 5. Overall, one can observe an improved accuracy of the predictors of the M-factor as the number of geometrical descriptors included in eqs 1–4 increases, despite the constants *c*_1_, *c*_2_, *c*_3_, *c*_4_ in these formulas being fitted in the literature for different materials and length scales. The most accurate predictors for the data in this study, as measured by MAPE and *R*^2^, are  for the CBD, and  for the pore space, see Table 5. Notably, for the CBD,  significantly outperforms the widely used predictor  which relies solely on ε.^50,64,66−68^ This is because two material phases with a similar volume fraction can still differ significantly in more sophisticated geometrical descriptors as shown in Section 3.1.

**Table 5:** Quantitative Comparison of Prediction Power of

Deviation measure	Phase				
MAPE	pore space	0.04181	0.04622	0.03616	0.03518
*R*^2^	pore space	0.83729	0.66458	0.80182	0.87475
MAPE	CBD	0.29574	0.16464	0.13955	0.16089
*R*^2^	CBD	0.68055	0.89553	0.91235	0.87457
Involved geometr. descriptors		ε	ε, β, μ(τ_g_)	ε, β, μ(τ_g_)	ε, μ(τ_g_), σ(τ_g_)

Moreover, due to the high porosity of samples SP30, SP45 and SP60, the prediction of the M-factor via  and  which both contain the constrictivity β, performs worse compared to  since bottleneck effects within the pore space seem not to be a limiting factor. For the CBD, using the standard deviation of geodesic tortuosity σ(τ_g_) instead of the constrictivity β leads to a significant improvement of the prediction accuracy. Notably, the predictor  given by the analytical regression formula in eq 4 is also the best predictor in (64) despite the study focusing on diffusion in loam and sand is considered at a much larger scale. This indicates that both the mean length of shortest transport paths and the variability of these lengths are suitable geometrical descriptors for predicting diffusive properties of multiphase materials, where the volume fraction of the transport phase is between 10 and 40%.

## 4 Conclusions and Outlook

In this contribution, we investigate three polymer-based battery electrodes, consisting of PTMA as active material, SuperP as the conductive additive and CMC as the binder, using 3D FIB-SEM tomography. The samples differ in material composition and, to the best of our knowledge, this is the first quantitative analysis of the three-dimensional morphology of polymer-based batteries at such a high-resolution. Our analysis shows a significant local heterogeneity within the electrode structure. The active material, synthesized via emulsion-polymerization, forms nanoparticles that tend to agglomerate during electrode processing from slurries. It turned out that the surface area of the active material is predominantly in contact with the pore space, which may hinder electron transport due to the comparatively low interface area between PTMA and the CBD, potentially reducing electrochemical performance. Among the three electrodes, the sample with the highest content of conductive additives has the finest structure as indicated by large surface areas and low mean chord lengths for both the pore space and the CBD. As in case of the geometrical descriptors, a strong local variation within the electrode is observed in the effective transport properties, which depend on microstructural features and influence electronic transport within the CBD and ionic transport through the pore space. Moreover, quantitative microstructure–property relationships are investigated. For the CBD, an analytical regression formula that includes the volume fraction, mean length of shortest transport paths and a bottleneck factor accurately predicts the M-factor. In particular, it is shown that the volume fraction of the CBD alone is not sufficient to predict the transport properties. In case of ionic transport within the pore space, the combination of porosity, mean length of shortest transport paths and the standard deviation of geodesic tortuosity, which quantifies the electrode’s local structural variability, allows reliable predictions of the M-factor. Regarding the prediction of effective electronic tortuosity of the CBD phase, the mean absolute percentage error is reduced by more than half by considering these more sophisticated geometrical descriptors instead of using the most widely used prediction formula that is solely based on the volume fraction. This highlights the importance of the electrode’s complex 3D geometry in influencing ionic transport.

In a forthcoming research paper, we aim to apply data-driven stochastic 3D microstructure modeling to generate a wide range of realistic virtual electrode structures, enabling virtual materials testing for polymer-based battery electrodes. In particular, the 3D FIB-SEM data considered in the present paper allows to take the morphology of nanopores into account, while synchrotron tomography will be used to quantify the large-scale spatial distribution of PTMA. The effective transport properties determined in this study can then be used to perform electrochemical simulations at the macroscopic scale. This will enable model-based investigations of the C-rate dependent power density, which - like the energy density - can be significantly affected by the electrode morphology. Another possible topic for future research is the investigation of the electrodes’s morphology after electrolyte filling and after cycling. To conclude, future research activities are required to exploit the full potential of polymer-based batteries with tailor-made microstructure as cost-effective and environmentally friendly energy storage technology.

## Data Availability

The data are available from the authors upon reasonable request.
